# Trends in *Mycoplasma pneumoniae* infections in pediatric patient preceding, during, and following the COVID-19 pandemic: a comprehensive longitudinal analysis

**DOI:** 10.1128/spectrum.01001-24

**Published:** 2025-01-14

**Authors:** Jianwei Zhang, Ruoya Wu, Liyan Mo, Jinlong Ding, Ke Huang

**Affiliations:** 1 Shaoxing Maternity and Child Health Care Hospital, Shaoxing, China; 2 Children’s Hospital, Zhejiang University School of Medicine, Hangzhou, China; City of Hope Department of Pathology, Duarte, California, USA

**Keywords:** COVID-19 pandemic, acute respiratory infection, *M. pneumoniae*, children

## Abstract

This study investigated the impact of the coronavirus disease 2019 (COVID-19) pandemic and its associated restrictive measures on *Mycoplasma pneumoniae* infections in children with acute respiratory tract infection. The study aimed to elucidate the epidemiological characteristics of *M. pneumoniae* infections before and during the pandemic and following the easing of restrictive measures. Pharyngeal secretions were collected from 1,0174 pediatric patients with acute respiratory infection (ARI) who were admitted to Shaoxing Maternity and Child Health Care Hospital (Shaoxing, China) between May 2018 and December 2023. The analysis concentrated on *M. pneumoniae* detection rates, epidemiological features, and clinical characteristics. Among 10,174 samples collected from 2018 to 2023, 1,060 strains of *M. pneumoniae* were detected, resulting in an overall positive rate of 10.42% (*n* = 1,060 out of 1,0176). Further breakdown revealed positive rates of 8.25% (*n* = 279 out of 3,381) in phase I (May 2018–December 2019), 2.40% (*n* = 87 out of 3,623) in phase II (January 2020–November 2022), and 21.89% (*n* = 694 out of 3,170) in phase III (December 2022–December 2023). In comparison to phase I, a noteworthy decline in the positive rate of *M. pneumoniae* was found during phase II, followed by a substantial increase during phase III (*P* < 0.05). Furthermore, the positive rate of *M. pneumoniae* exhibited a significantly greater surge during the summer-autumn period in contrast to the winter-spring (*P* < 0.05). Additionally, the risk of *M. pneumoniae* infection in children who aged 7 years surpassed that of the age group of 0–3 years. Throughout the phases preceding, during, and after the COVID-19 pandemic, discernible fluctuations in both age distribution and seasonality of *M. pneumoniae* infections were identified among hospitalized children with ARI in the East China.

**IMPORTANCE:**

The resemblance between the transmission pathways of *M. pneumoniae* and those of COVID-19 suggests that non-pharmaceutical interventions may have comparable effects on both. This study aimed to analyze the epidemiological characteristics of children with *M. pneumoniae* infections in Shaoxing, China, from 2018 to 2023. This study explored how the COVID-19 pandemic has influenced the prevalence of *M. pneumoniae* and provided guidance for disease treatment and infection prevention.

## INTRODUCTION

Acute respiratory infection (ARI) is the fourth leading cause of global mortality, with pneumonia and bronchiolitis emerging as the predominant reasons for pediatric hospitalization and in-hospital fatalities (1). *Mycoplasma pneumoniae* is recognized as a significant pathogen contributing to ARI in children, particularly with more than 10% of cases progressing to pneumonia (2). School-age children and adolescents exhibited heightened susceptibility to *M. pneumoniae* infection, with prevalence rates ranging from 10% to 40% in pneumonia cases (3).

Transmission of *M. pneumoniae* primarily occurs through respiratory droplets, thriving in enclosed settings such as schools, childcare institutions, and summer camps. The infection follows an endemic pattern characterized by periodic peaks every few years. National surveillance in China reported a cumulative incidence of 18.6% for ARI from 2009 to 2019 (1). Globally, the incidence of *M. pneumoniae* infection was 8.61% between 2017 and 2020. However, during the coronavirus disease 2019 (COVID-19) pandemic (2020–2021), non-pharmaceutical interventions (NPIs) led to a remarkable decline, reducing the incidence to 1.69% (4). Despite the relaxation or discontinuation of NPIs by 2022, a sustained reduction in incidence persisted across 20 countries in Europe, Asia, the Americas, and Oceania (5).

Between January 2020 and early December 2022, China implemented the world’s most stringent and enduring restrictive measures to combat the pandemic. The prolonged use of these NPIs might result in an “immune debt,” potentially overprotecting children. Upon lifting restrictions, the possibility of intensified epidemics caused by respiratory pathogens, such as respiratory syncytial virus and influenza virus, arises. The majority of children, especially infants and preschool, may constitute a sizable susceptible population due to limited exposure to these respiratory pathogens. Notably, in 2023, China experienced an epidemic peak of *M. pneumoniae*, commencing in April and peaking from October to November (6).

The available data concerning the epidemiological characteristics of *M. pneumoniae* in children at different stages of the COVID-19 pandemic in China are currently limited. In response, pertinent data on mycoplasma infections were systematically extracted from the hospital’s laboratory information system. This retrospective study was conducted to evaluate variations in *M. pneumoniae* infections in children, covering the periods before, during, and after the COVID-19 pandemic, including the post-restriction phase. This study may provide evidence-based insights, facilitating the formulation of strategic approaches for the prevention of *M. pneumoniae* infections in the pediatric population.

## MATERIALS AND METHODS

### Study population

To explore the characteristics of *M. pneumoniae* infection in children before and during the COVID-19 pandemic and subsequent to the relaxation of COVID-19 restrictions, this retrospective analysis was conducted. The investigation concentrated on pediatric cases of ARI admitted to Shaoxing Maternity and Child Health Hospital (Class III Grade I Maternity and Child Health Hospital in East China), spanning the period from May 2018 to December 2023. A total of 10,174 children diagnosed with ARI were included in the study. A senior pediatrician precisely carried out the diagnosis. Exclusion criteria for children with ARI were established to refine the study cohort. The reasons underlying patients’ exclusion were summarized as follows: (i) multiple hospital visits within a single week; (ii) instances of hospital-acquired infection; (iii) the presence of congenital pulmonary airway malformation or immune system impairment, congenital heart disease, or other underlying medical conditions. While the primary concentration of this study was the detection of *M. pneumoniae* through PCR, future studies may benefit from incorporating genotyping and macrolide resistance analysis. This will provide additional insights into the evolution of circulating *M. pneumoniae* strains and their resistance profiles, which have been noted in similar investigations of respiratory pathogens.

### Data collection

Respiratory tract samples were collected from all eligible inpatients below 15 years of age diagnosed with ARI. A qualified nurse conducted the data collection via a throat swab within the initial 24 h of hospitalization. Utilizing sterile flocked nylon swabs, samples were obtained from the bilateral pharyngeal tonsils and the posterior pharyngeal wall. These swabs were subsequently carefully placed into sterile tubes containing 2 mL of sterile saline. The tightly packed test tubes were promptly sent to the hospital’s laboratory medicine department for timely testing. Detection of *M. pneumoniae* was carried out using the PCR-fluorescent probe method with six respiratory pathogen nucleic acid detection kits (Sansure Biotech Inc., Shanghai, China) (7).

### Detection method

For nucleic acid extraction, 200 µL of the samples, *M. pneumoniae* negative control, or positive control was combined in a 1.5 mL centrifuge tube containing nucleic acid extraction reagent (Sansure Biotech Inc.). Subsequently, 5 µL of the treated samples, positive control, and negative control were added to the corresponding 0.2 mL PCR reaction tube. Each tube received 45 µL of the PCR mixture, with the cap tightly secured. The processed samples were then tested using a designated sample point board. PCR reaction tubes were placed in the amplification instrument’s sample tank; fluorescence detection channels were selected; and cycle parameters were configured. The post-reaction results were automatically saved and analyzed. A clear S-type amplification curve with a Ct value of ≤40 indicated a positive result, while the absence of an amplification curve or a Ct value of >40 was considered negative.

To facilitate data analysis, seasons were categorized as follows: winter (December–February), spring (March–May), summer (June–August), and autumn (September–November). The study period was divided into three phases: phase I (May 2018–December 2019, pre-pandemic period of COVID-19), phase II (January 2020–November 2022, pandemic prevention and control period of COVID-19), and phase III (December 2022–December 2023, pandemic prevention and control remission period of COVID-19).

### Statistical analysis

R version 4.3.1 language processing served as the analytical tool for this study. A normality test was conducted to assess the normality of continuous variables, such as age. The positive detection rate of *M. pneumoniae*, along with its 95% confidence interval, in children with ARI was computed based on gender, age, season, and the three defined stages of the COVID-19 epidemic. Group comparisons were conducted using *χ*^2^ test or Fisher’s exact probability method as appropriate, exploring associations between *M. pneumoniae*-positive detection rates and gender, age, season, and the distinct phases of the epidemic. The non-linear correlation analysis between age and the positive detection rate was assessed using a restricted cubic spline regression model with four nodes strategically positioned at the 5th, 25th, 75th, and 95th percentiles. A significance level of *P* < 0.05 was established to denote statistical significance, guiding the interpretation of findings and highlighting associations that warrant attention in the context of *M. pneumoniae* infection among children with ARI.

## RESULTS

### Overall detection rate of *M. pneumoniae* infection in children with ARI

Among the 10,174 children with ARI who were enrolled in this study, 5,888 were boys and 4,286 were girls. The age distribution exhibited a median of 2.1 (0.4–3.0 years), with 2,041 cases in spring, 2,551 in summer, 3,025 in autumn, and 2,557 in winter. Across the three defined stages of the study period, there were 3,381 cases in the first stage, 3,887 in the second stage, and 2,906 in the third stage. Notably, there were no statistically significant differences in age and gender among children across these stages. A comprehensive overview of the demographic characteristics is presented in Table 1. The total positive detection rate of *M. pneumoniae* in children with ARI was 10.42% (9.82%–11.01%). Stratifying by stages, the positive detection rates were 8.25% (7.32%–9.18%) in the first stage, 2.40% (1.90%–2.90%) in the second stage, and 21.89% (20.45%–23.33%) in the third stage. A significant difference in the overall positive detection rate was observed among the three stages (*χ*^2^ = 713.62, *P* < 0.001). Further analysis indicated that the positive detection rate of *M. pneumoniae* in boys was 11.48% (10.52%–12.43%), while that in girls was 9.4% (8.89%–10.40%). Notably, the infection rate of *M. pneumoniae* was found to be higher in boys compared with that in girls (*P* = 0.005), as detailed in Table 2.

**TABLE 1:** General and clinical characteristics of the study population

	Total, *n* (%)	Phase I, *n* (%)	Phase II, *n* (%)	Phase III, *n* (%)	*χ* ^2^	*P* value
		(May 2018–December)	(January 2020–November 2022)	(December 2022–December 2023)		
Overall	1,0174	3,381 (33.23)	3,623 (35.61)	3,170 (31.16)	13.682	<0.001
Age (years)					479.248	<0.001
0–1	4,187 (41.15)	1,588 (46.97)	1,611 (44.47)	988 (31.17)		
1–3	2,757 (27.10)	945 (27.95)	1,010 (27.88)	802 (25.30)		
3–7	2,610 (25.65)	759 (22.45)	864 (23.85)	987 (31.14)		
7–14	620 (6.09)	89 (2.63)	138 (3.81)	393 (12.40)		
Gender					6.957	0.031
Female	4,286 (42.13)	1,390 (41.11)	1,500 (41.40)	1,396 (44.04)		
Male	5,888 (57.87)	1,991 (58.89)	2,123 (58.60)	1,774 (55.96)		
Season					52.995	<0.001
Winter	2,557 (25.13)	784 (23.19)	932 (25.72)	841 (26.53)		
Spring	2,041 (20.06)	621 (18.37)	820 (22.63)	600 (18.93)		
Summer	2,551 (25.07)	962 (28.45)	816 (22.52)	773 (24.38)		
Autumn	3,025 (29.73)	1,014 (29.99)	1,055 (29.12)	956 (30.16)		
Outcome					713.682	<0.001
Negative	9,114 (89.58)	3,102 (30.49)	3,536 (34.76)	2,476 (24.34)		
Positive	1,060 (10.42)	279 (2.74)	87 (0.86)	694 (6.82)		

**TABLE 2:** Positive detection rates and 95% confidential intervals of *M. pneumoniae* infection across age groups

	Total	Phase I (May 2018–December 2019)	Phase II (January 2020–November 2022)	Phase III (December 2022–December 2023)	X^2^	*P* value
Overall	10.42 (9.82–11.01)	8.25 (7.32–9.18)	2.40 (1.90–2.90)	21.89 (20.45–23.33)	713.62	<0.001
Age (years)					96.456	<0.001
0–1	3.39 (2.84–3.94)	4.35 (3.34–5.35)	0.99 (0.51–1.48)	5.77 (4.31–7.23)		
1–3	7.98 (6.97–8.99)	8.47 (6.69–10.24)	2.08 (1.20–2.96)	14.84 (12.37–17.30)		
3–7	16.44 (15.01–17.86)	13.44 (11.01–15.87)	2.89 (1.77–4.01)	30.60 (27.72–33.48)		
7–14	43.39 (39.48–47.30)	31.46 (21.62–40.30)	18.12 (11.61–24.62)	54.96 (50.02–59.90)		
*P* value	0.000	0.000	0.000	0.000		
Gender					5.401	<0.001
Female	11.48 (10.52–12.43)	8.13 (6.69–9.57)	2.73 (1.91–3.56)	24.21 (21.96–26.46)		
Male	9.64 (8.89–10.40)	8.34 (7.12–9.55)	2.17 (1.55–2.79)	20.07 (18.20–21.93)		
*P* value	0.005	0.829	0.273	0.005		
Season					58.593	<0.001
Winter	7.39 (6.40–8.44)	5.74 (4.11–7.37)	1.29 (0.56–2.01)	15.70 (13.23–18.16)		
Spring	5.29 (4.32–6.26)	7.09 (5.06–9.11)	1.95 (1.00–2.90)	8.00 (5.82–10.18)		
Summer	13.68 (12.35–15.02)	12.16 (10.09–14.23)	4.29 (2.90–5.68)	25.49 (22.41–28.56)		
Autumn	13.69 (12.46–14.91)	7.20 (5.61–8.79)	2.27 (1.37–3.18)	33.16 (30.17–36.15)		
*P* value	0.000	0.000	0.000	0.000		

### Age distribution of *M. pneumoniae* infection in children with ARI

Within the cohort of 10,174 children experiencing ARI, age distribution revealed 4,187 cases in the age group of 0–1 year; 2,757 cases in the age group of 1–3 years; 2,610 cases in the age group of 3–7 years; and 620 cases in the age group of 7–14 years. Strikingly, the highest positive rate of *M. pneumoniae* infection was found in the age group of 7–14 years, reaching 43.39% (39.4%–47.30%). This was followed by the age group of 3–7 years, with a positive rate of 16.44% (15.01%–17.86%). Conversely, the age group of 0–1 year exhibited the lowest positive rate at 3.39% (2.84%–3.94%), revealing a statistically significant difference across the age groups (*P* = 0.000). Remarkably, irrespective of the temporal context, whether before the COVID-19 pandemic, during its occurrence, or after the relaxation of prevention and control measures, the age group of 7–14 years consistently demonstrated the highest positive detection rate for *M. pneumoniae* in children with ARI. Conversely, the age group of 0–1 year consistently exhibited the lowest detection rate. A detailed breakdown of these findings is presented in Table 2.

### Seasonal and monthly distribution of *M. pneumoniae* infection in children with ARI

**Fig 1:**
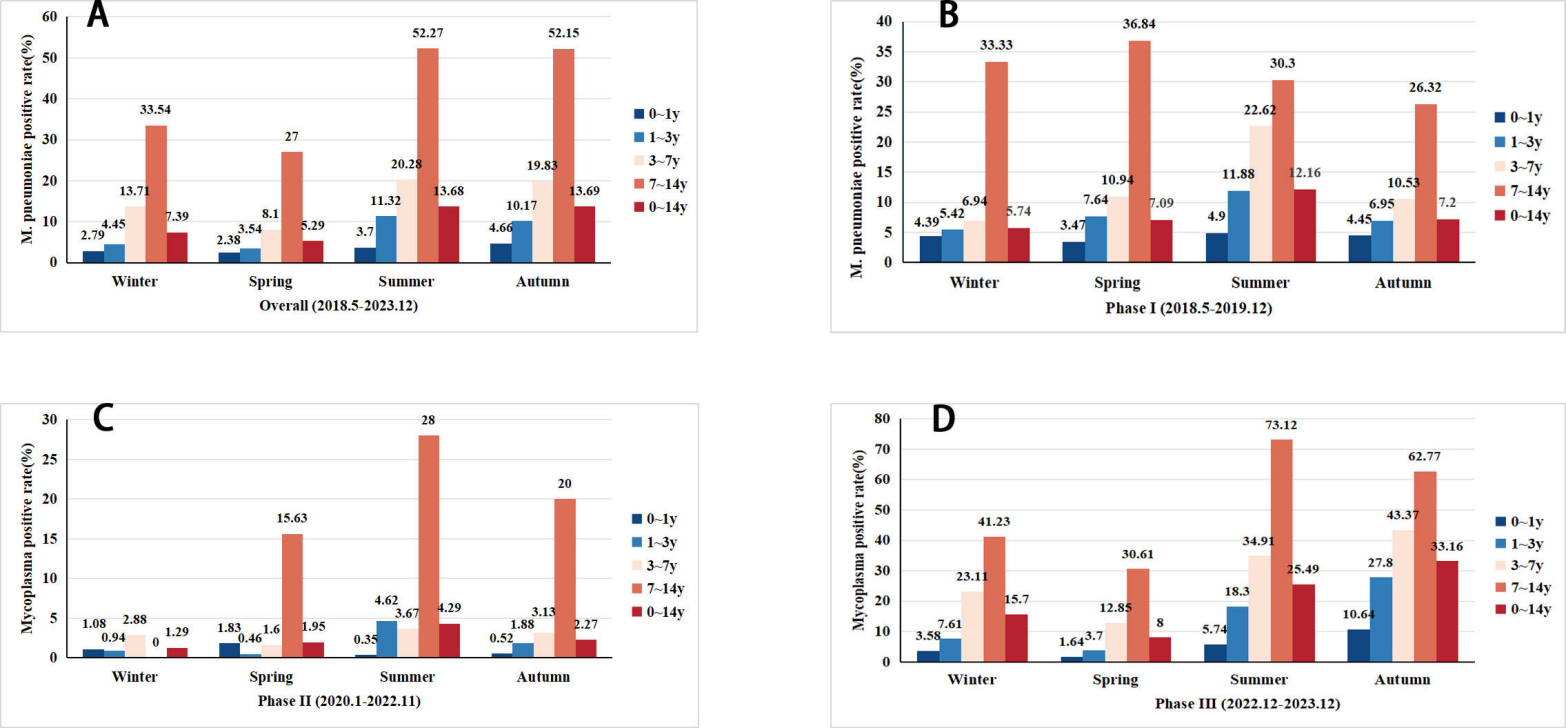
Positive detection rates of *Mycoplasma pneumoniae* across different age groups (0–14 years) and seasons (winter, spring, summer, and autumn) during three phases of the COVID-19 pandemic. (**A**) Overall detection rates across all patients from May 2018 to December 2023. The highest positivity rates were observed in summer and autumn, predominantly in the age group of 0–14 years. (**B**) Detection rates before the COVID-19 pandemic (phase I: May 2018–December 2019). Higher positivity rates were seen in winter and spring, especially in the age group of 0–14 years. (**C)** Detection rates during the COVID-19 pandemic (phase II: January 2020–November 2022). The rates dropped overall, with a slight peak in summer in the age group of 0–14 years. (**D)** Detection rates after the easing of COVID-19 restrictions (phase III: December 2022–December 2023). A sharp increase in positivity was observed in summer and autumn, particularly in the age group of 0–14 years.

In this study encompassing 10,174 samples, the positive detection rate of *M. pneumoniae* varied across seasons: winter was 7.39% (6.40%–8.44%); spring was 5.29% (4.32%–6.26%); summer was 13.68% (12.35%–15.02%); and autumn was 13.69% (12.46%–14.91%). The highest detection rate was observed during summer-autumn, while the lowest occurred in spring, revealing a significant seasonal difference (*P* = 0.000). Figure 1 illustrates the positive detection rate of *M. pneumoniae* in different seasons and age groups during each stage of the COVID-19 pandemic. During the first and the third stages, the positive detection rate of *M. pneumoniae* increased with age. In contrast, an intriguing shift was found in the second stage, with the age group of 3–7 years exhibiting the highest positive detection rate in winter. Simultaneously, in the spring of the second stage, an intriguing shift emerged, showcasing a higher positive detection rate of *M. pneumoniae* in the age group of 0–1 year compared with the age group of 1–7 years.

In the first stage of the COVID-19, the highest positive detection rate of *M. pneumoniae* was 12.16% (10.09%–14.23%) in summer, and the lowest was 5.74% (4.11%–7.37%) in winter. In the second stage, the highest positive detection rate was 4.29% (2.90%–5.68%) in summer, and the lowest rate was 1.29% (0.56%–2.01%) in winter. In the third stage, the highest positive detection rate was 33.16% (30.17%–36.15%) in autumn, while the lowest rate was 0.08% (5.82%–10.18%) in spring.

**Fig 2:**
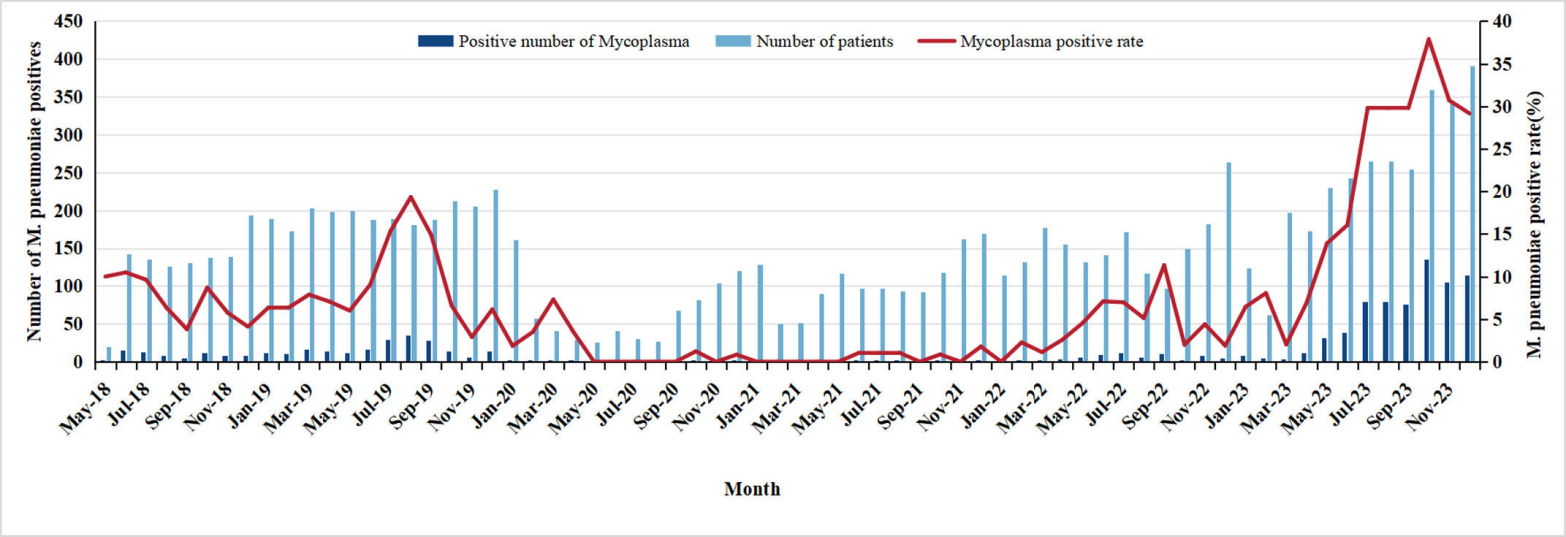
Monthly positive detection rates of *Mycoplasma pneumoniae* from May 2018 to December 2023. The bar graph represents the total number of patients tested each month (light blue bars), while the dark blue bars show the number of patients who tested positive for *M. pneumoniae*. The red line represents the positive detection rate (%) of *M. pneumoniae*. A notable peak in positive cases and detection rate occurred around late 2023, following a low detection phase during 2020–2021, corresponding with the COVID-19 pandemic.

Figure 2 illustrates the positive detection rate of *M. pneumoniae* in each month from May 2018 to December 2023. The trajectory of *M. pneumoniae* infection in children reveals distinct patterns, resembling a sequence of “seesawing,” “off-season,” another seesawing phase, and culminating in an “upsurge.” Notably, the infection rate exhibited a gradual rise starting from May 2019, with a minor peak in September of the year. Subsequently, a significant decrease ensued, reaching the lowest point in May 2020. Following this trough, a gradual increase commenced from May 2022, accelerating into a rapid upward trend in April 2023, ultimately peaking in the autumn.

### Non-linear relationship between age and positive detection rate of *M. pneumoniae*

**Fig 3:**
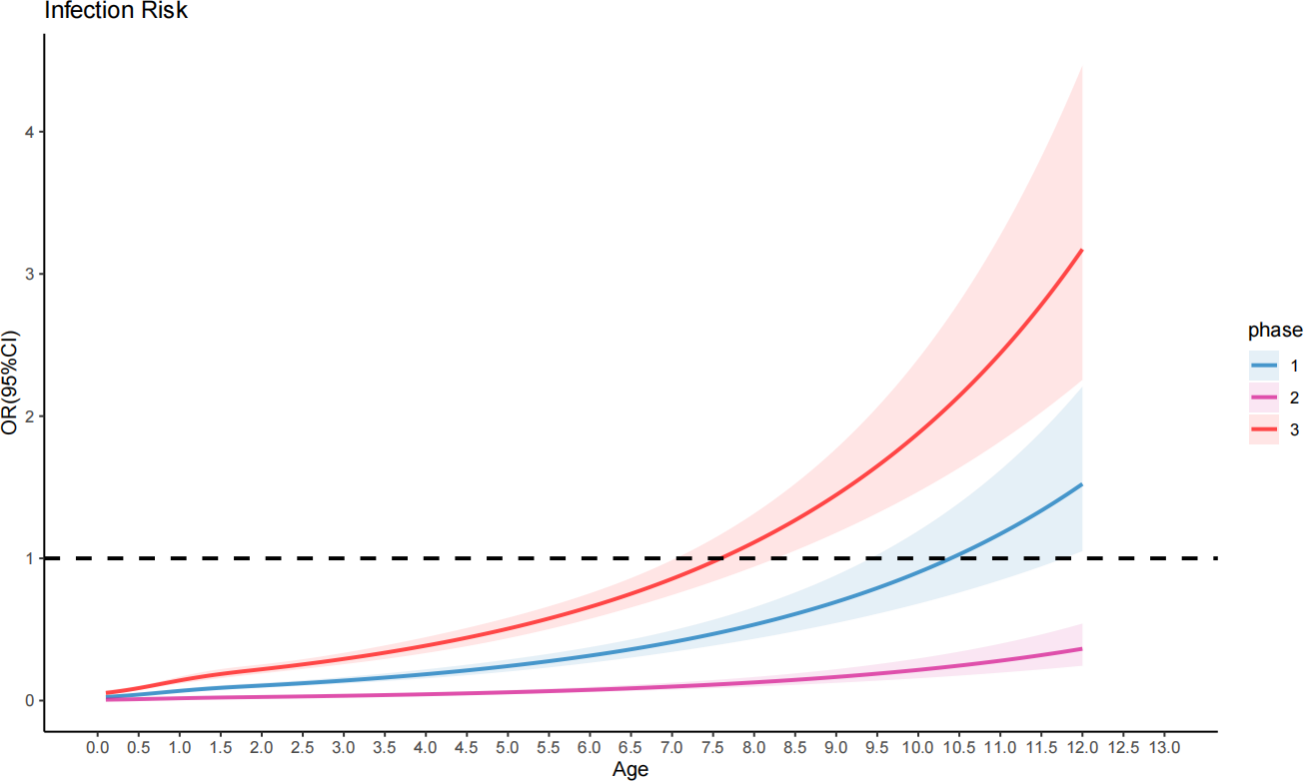
Non-linear association between age and risk of *Mycoplasma pneumoniae* infection. The non-linear relationship between age and the risk of *M. pneumoniae* infection, as estimated by restricted cubic spline regression models, is illustrated. The estimated infection risk is shown on the *Y*-axis, and the age in years is shown on the *X*-axis. The shaded areas around each curve represent the 95% confidence intervals (CIs), indicating the uncertainty of the estimates at different ages. Phase I: pre-pandemic period of COVID-19, phase II: pandemic prevention and control period of COVID-19, phase III: pandemic prevention and control remission period of COVID-19. All models show statistically significant overall associations between age and infection risk (*P* < 0.001), with the non-linear components also being significant (*P* < 0.001).

Analyzing the non-linear relationship between age and the positive detection rate of *M. pneumoniae*, we employed a cubic regression model in this study for detailed examination at each stage of the COVID-19 pandemic (Fig. 3). We found that the odds ratio (OR) for children was low ( <1) in the second stage. In the first stage, the OR for children over 10.4 years old was >1, and in the third stage, patients aged around 7.6 years old exhibited an OR of >1. The risk of *M. pneumoniae* infection increased with age in children in the first and third stages.

## DISCUSSION

In this comprehensive study, we analyzed *M. pneumoniae* infections in 10,174 infants, children, and adolescents aged 0–14 years and hospitalized for ARI during three distinct stages of the COVID-19 pandemic (May 2018–December 2023). To our knowledge, this constitutes the first extensive analysis of *M. pneumoniae* infection in children with ARI at different stages of the COVID-19 pandemic in China. Notably, the overall positive detection rate of *M. pneumoniae* in children with ARI in our study, though lower than the reported 48.4%–61.1% in Beijing, China (8), aligns closely with the 11.51% infection rate reported in South Korea over the past 5 years (9).

The findings also revealed a pre-pandemic positive detection rate of *M. pneumoniae* at 8.5%, with a minor epidemic peak in October 2019, consisting of Zhang et al.’s findings (10). This aligns with the globally recognized unified epidemic model for *M. pneumoniae* infection (11). During the pandemic, there was a substantial decrease in *M. pneumoniae* infection, followed by a noteworthy surge after the relaxation of prevention and control measures. The robust pandemic prevention and control measures significantly curtailed community-acquired pneumonia attributed to *M. pneumoniae* infection after the onset of the COVID-19 epidemic (12). Stringent measures were implemented in Shaoxing on 23 January 2020, which closely resembled those implemented to curb the spread of the novel coronavirus. Both *M. pneumoniae* and COVID-19 share a similar transmission route, primarily through droplet transmission in close contact, indicating the comparable effectiveness of non-drug interventions (13). The present research substantiates this correlation, demonstrating a rapid decline in the positive detection rate of *M. pneumoniae* in the general population from March 2020, maintaining a consistently low level from May to October. Notably, the resurgence of *M. pneumoniae* infection in children commenced in May 2021. The hypothesis suggests that as the COVID-19 epidemic subsided and pandemic management became more routine, individuals in low-risk areas, particularly parents, resumed pre-pandemic work and lifestyle patterns. This shift created favorable conditions for the re-emergence and spread of *M. pneumoniae*.

In general, *M. pneumoniae* infections tend to follow an epidemic cycle of 3–5 years, a phenomenon documented in the literature (14, 15). The subtle alterations in population-level immunity duration may play a pivotal role in shaping the cyclical nature of *M. pneumoniae* outbreaks (16). As of mid-December 2022, China has undergone a comprehensive easing of nationwide COVID-19 restrictions, marking the commencement of a new phase in the post-epidemic era from 2023. Notably, *M. pneumoniae* infections exhibited a distinct “rapid rise” pattern during this period, a trend potentially linked to the concept of immune debt. Immune debt is defined as a deficiency in protective immunity, rendering individuals more susceptible to infectious diseases (17). The stringent preventive measures implemented during the COVID-19 epidemic may result in children’s limited exposure to various infectious pathogens, potentially impacting their immune development. This observation aligns with Bai et al.’s findings, who reported immune debt in the context of the RSV epidemic during the novel coronavirus outbreak (18). Additionally, some researchers pointed out that the peak of *M. pneumoniae* infections in China in 2023 could be influenced by the genetic instability of the strains (8).

*M. pneumoniae* infections are known to manifest in any season, exhibiting diverse epidemic patterns across different regions of China (19). In the present study, the prevalence of *M. pneumoniae* infection exhibited a distinct seasonal variation. The infection peaked during the summer and persisted during the new coronavirus epidemic, whereas after the relaxation of prevention and control measures, the peak shifted to autumn. *M. pneumoniae* manifested most effectively at temperatures between 35°C and 37°C, suggesting a greater tendency for growth and transmission in warmer conditions. Shaoxing, located at 120° east longitude and 30° north latitude, experiences long hours of sunshine during summer and autumn, with average temperatures between 35°C and 37°C, which is within the optimal range for *M. pneumoniae* survival. Notably, despite the gradual increase in regional temperatures from May 2020, the positive detection rate of *M. pneumoniae* experienced a decline, reaching an exceptionally low level from May to October. This anti-seasonal phenomenon may be attributed to the rigorous measures imposed to limit the spread of the new coronavirus.

Moreover, the present study corroborates the observation that *M. pneumoniae* infections primarily occur in children over 5 years old (20). Regardless of the pandemic stage, the findings consistently highlight that *M. pneumoniae* infections are most prevalent in older children in China. As age advances, the positive detection rate of *M. pneumoniae* increases gradually, aligning with previous research outcomes (21). The study of the non-linear relationship between age and the risk of *M. pneumoniae* infection throughout the three stages of the COVID-19 pandemic revealed that the risk of *M. pneumoniae* infection was at its lowest during the pandemic, undergoing a dramatic escalation following the relaxation of restrictive measures. In addition, the data also show that in the absence of epidemic prevention and control measures, the risk of *M. pneumoniae* infection increases with age. Notably, after the epidemic, the age of susceptibility has advanced, suggesting that epidemic prevention and control measures are also effective in preventing *M. pneumoniae* infection. This suggests a notable vulnerability in the population of all children, emphasizing the significance of these findings for public health and clinical practice.

Although this study concentrated on the epidemiological characteristics of *M. pneumoniae* infection, future research will incorporate detailed information on *M. pneumoniae* genotypes and macrolide resistance patterns. This will provide a more comprehensive understanding of the potential shifts in strain characteristics and antibiotic resistance trends over time, especially in the context of the COVID-19 pandemic, as previously reported in similar studies.

This study represents a notable contribution, providing a comprehensive examination of the positive detection rate of *M. pneumoniae* with a substantial sample size and an extended observation period, strategically utilizing the new coronavirus epidemic as a pivotal intermediate time node. To the best of our knowledge, this study represents the first evaluation project of its kind in China. The advanced application of the restricted cubic spline regression model in assessing the non-linear relationship between age and *M. pneumoniae* infection, tailored to the age-specific characteristics of *M. pneumoniae* infection, emphasizes the methodological strength of this study’s approach. However, certain limitations are noteworthy. Firstly, this study is singularly centered, potentially introducing bias, especially given the relatively lower inclusion of children over 10 years old, which might compromise statistical power. Thus, we advocate for future multi-center studies to corroborate or challenge our findings. Secondly, this research lacked a precise diagnosis of the *M. pneumoniae* genotype, an omission that could hold clinical implications, as different subtypes of *M. pneumoniae* may carry different clinical significance.

In conclusion, this study provided valuable insights into the positive detection of *M. pneumoniae* in children with ARI at different stages of the COVID-19 pandemic in Shaoxing, eastern China, shedding light on the epidemiological characteristics of *M. pneumoniae* infection in children. The susceptibility of older children to infection is notably underscored, particularly with the relaxation of COVID-19 restrictions, leading to a peak in the prevalence of *M. pneumoniae* infection. Health professionals and nursing staff are encouraged to remain vigilant and attentive to the changing epidemiological patterns of *M. pneumoniae* infection in the post-epidemic period, particularly following the relaxation of restrictions.

## References

[1] Li Z-J, Zhang H-Y, Ren L-L, Lu Q-B, Ren X, Zhang C-H, Wang Y-F, Lin S-H, Zhang X-A, Li J, et al.. 2021. Etiological and epidemiological features of acute respiratory infections in China. Nat Commun 12:5026. doi:10.1038/s41467-021-25120-6 34408158 PMC8373954

[2] Krafft C, Christy C. 2020. Mycoplasma pneumonia in children and adolescents. Pediatr Rev 41:12–19. doi:10.1542/pir.2018-0016 31894069

[3] Li L, Ma J, Guo P, Song X, Li M, Yu Z, Yu Z, Cheng P, Sun H, Zhang W. 2022. Molecular beacon based real-time PCR p1 gene genotyping, macrolide resistance mutation detection and clinical characteristics analysis of Mycoplasma pneumoniae infections in children. BMC Infect Dis 22:724. doi:10.1186/s12879-022-07715-6 36068499 PMC9447981

[4] Meyer Sauteur PM, Beeton ML, Uldum SA, Bossuyt N, Vermeulen M, Loens K, Pereyre S, Bébéar C, Keše D, Day J, Afshar B, Chalker VJ, Greub G, Nir-Paz R, Dumke R, ESGMAC–MyCOVID Study Team. 2022. Mycoplasma pneumoniae detections before and during the COVID-19 pandemic: results of a global survey, 2017 to 2021. Euro Surveill 27:2100746. doi:10.2807/1560-7917.ES.2022.27.19.2100746 35551702 PMC9101966

[5] Meyer Sauteur PM, Chalker VJ, Berger C, Nir-Paz R, Beeton ML, ESGMAC and the ESGMAC–MyCOVID study group. 2022. Mycoplasma pneumoniae beyond the COVID-19 pandemic: where is it? Lancet Microbe 3:e897. doi:10.1016/S2666-5247(22)00190-2 35964636 PMC9371584

[6] Zhang X-B, He W, Gui Y-H, Lu Q, Yin Y, Zhang J-H, Dong X-Y, Wang Y-W, Ye Y-Z, Xu H, Wang J-Y, Shen B, Gu D-P, Wang L-B, Wang Y. 2024. Current Mycoplasma pneumoniae epidemic among children in Shanghai: unusual pneumonia caused by usual pathogen. World J Pediatr 20:5–10. doi:10.1007/s12519-023-00793-9 38231466

[7] Wang M, Wang Y, Yan Y, Zhu C, Huang L, Shao X, Xu J, Zhu H, Sun X, Ji W, Chen Z. 2014. Clinical and laboratory profiles of refractory Mycoplasma pneumoniae pneumonia in children. Int J Infect Dis 29:18–23. doi:10.1016/j.ijid.2014.07.020 25449230

[8] Yan C, Xue G-H, Zhao H-Q, Feng Y-L, Cui J-H, Yuan J. 2024. Current status of Mycoplasma pneumoniae infection in China. World J Pediatr 20:1–4. doi:10.1007/s12519-023-00783-x 38185707 PMC10827902

[9] Shin S, Koo S, Yang Y-J, Lim H-J. 2023. Characteristics of the Mycoplasma pneumoniae epidemic from 2019 to 2020 in Korea: macrolide resistance and co-infection trends. Antibiotics (Basel) 12:1623. doi:10.3390/antibiotics12111623 37998825 PMC10669541

[10] Zhang Y, Huang Y, Ai T, Luo J, Liu H. 2021. Effect of COVID-19 on childhood Mycoplasma pneumoniae infection in Chengdu, China. BMC Pediatr 21:202. doi:10.1186/s12887-021-02679-z 33910509 PMC8079841

[11] Beeton ML, Zhang X-S, Uldum SA, Bébéar C, Dumke R, Gullsby K, Ieven M, Loens K, Nir-Paz R, Pereyre S, Spiller OB, Chalker VJ, ESCMID Study Group for Mycoplasma and Chlamydia Infections (ESGMAC) Mycoplasma pneumoniae subgroup, ESCMID Study Group for Mycoplasma and Chlamydia Infections (ESGMAC) Mycoplasma pneumoniae subgroup members not listed as an individual author. 2020. Mycoplasma pneumoniae infections, 11 countries in Europe and Israel, 2011 to 2016. Euro Surveill 25:1900112. doi:10.2807/1560-7917.ES.2020.25.2.1900112 31964459 PMC6976882

[12] Zhang L-N, Cao L, Meng L-H. 2022. Pathogenic changes of community-acquired pneumonia in a children’s hospital in Beijing, China before and after COVID-19 onset: a retrospective study. World J Pediatr 18:746–752. doi:10.1007/s12519-022-00592-8 35994171 PMC9395926

[13] Steinberg P, White RJ, Fuld SL, Gutekunst RR, Chanock RM, Senterfit LB. 1969. Ecology of Mycoplasma pneumoniae infections in marine recruits at Parris Island, South Carolina. Am J Epidemiol 89:62–73. doi:10.1093/oxfordjournals.aje.a120916 5812542

[14] Lenglet A, Herrador Z, Magiorakos AP, Leitmeyer K, Coulombier D, European Working Group on Mycoplasma pneumoniae surveillance. 2012. Surveillance status and recent data for Mycoplasma pneumoniae infections in the European Union and European Economic Area, January 2012. Euro Surveill 17:20075. doi:10.2807/ese.17.05.20075-en 22321134

[15] Kurkela S, Puolakkainen M, Hokynar K, Nieminen T, Saxen H, Mannonen L, Pietikäinen R. 2019. Mycoplasma pneumoniae outbreak, Southeastern Finland, 2017-2018: molecular epidemiology and laboratory diagnostic lessons. Eur J Clin Microbiol Infect Dis 38:1867–1871. doi:10.1007/s10096-019-03619-7 31263967 PMC6778538

[16] Omori R, Nakata Y, Tessmer HL, Suzuki S, Shibayama K. 2015. The determinant of periodicity in Mycoplasma pneumoniae incidence: an insight from mathematical modelling. Sci Rep 5:14473. doi:10.1038/srep14473 26412506 PMC4585982

[17] Yang M-C, Su Y-T, Chen P-H, Tsai C-C, Lin T-I, Wu J-R. 2023. Changing patterns of infectious diseases in children during the COVID-19 pandemic. Front Cell Infect Microbiol 13:1200617. doi:10.3389/fcimb.2023.1200617 37457965 PMC10339349

[18] Hu L, Yang Y, Lin J, Yan Q, Sun C, Li Z, Sun L, Xu J, Chen J, Bai G. 2024. Epidemiological characteristics of respiratory syncytial virus infection in pediatric patients before, during the COVID-19 pandemic and after easing of COVID-19 restrictive measures in China. J Med Virol 96:e29374. doi:10.1002/jmv.29374 38197487

[19] Wang X, Li M, Luo M, Luo Q, Kang L, Xie H, Wang Y, Yu X, Li A, Dong M, Huang F, Gong C. 2022. Mycoplasma pneumoniae triggers pneumonia epidemic in autumn and winter in Beijing: a multicentre, population-based epidemiological study between 2015 and 2020. Emerg Microbes Infect 11:1508–1517. doi:10.1080/22221751.2022.2078228 35582916 PMC9176688

[20] Atkinson TP, Waites KB. 2014. Mycoplasma pneumoniae infections in childhood. Pediatr Infect Dis J 33:92–94. doi:10.1097/INF.0000000000000171 24346598

[21] Li X, Li T, Chen N, Kang P, Yang J. 2023. Changes of Mycoplasma pneumoniae prevalence in children before and after COVID-19 pandemic in Henan, China. J Infect 86:256–308. doi:10.1016/j.jinf.2022.12.030 PMC9838080 36646141

